# Kick the Cat: A Serial Crossover Effect of Supervisors’ Ego Depletion on Subordinates’ Deviant Behavior

**DOI:** 10.3389/fpsyg.2020.01314

**Published:** 2020-06-11

**Authors:** Xiaodong Ming, Xinwen Bai, Lin Lin

**Affiliations:** ^1^CAS Key Laboratory of Behavioral Science, Institute of Psychology, Chinese Academy of Sciences, Beijing, China; ^2^Department of Psychology, University of Chinese Academy of Sciences, Beijing, China; ^3^School of Business, Central University of Finance and Economics, Beijing, China

**Keywords:** ego depletion, abusive supervision, unethical behavior, crossover, conservation of resources

## Abstract

Drawing on the crossover model and conservation of resources theory, we explore the mechanism through which supervisors’ ego depletion induces subordinates’ deviant behavior. Using the two-wave survey data from 24 supervisors and their 192 respective subordinates, we found supports for our hypotheses that (a) abusive supervision mediated the effect of supervisors’ ego depletion on subordinates’ ego depletion; (b) subordinates’ ego depletion mediated the effect of abusive supervision on subordinates’ deviant behavior; and (c) abusive supervision and subordinates’ ego depletion serially mediated the effect of supervisors’ ego depletion on subordinates’ deviant behavior. Our serial crossover model posits that both ego depletion and unethical behavior can be transmitted from supervisors to subordinates, and that these two crossover processes are entwined with each other. Findings are discussed in terms of theoretical contributions and practical implications.

“It is the way of the world, Baldrick. The abused always kick downwards. I am annoyed, and so I kick the cat, the cat pounces on the mouse, and finally, the mouse bites you on the behind.”
  —Blackadder (Lloyd, 1987)

## Introduction

Individuals are easily falling into the state of ego depletion in organizations due to numerous work stresses (Lian et al., 2017). Different from negative emotional experiences (e.g., burnout, emotional exhaustion, and anxiety), ego depletion refers to a cognitive state of diminished self-control resources (Baumeister and Vohs, 2016). Under an ego depletion state, individuals lack sufficient self-control resources to control themselves to do volitional behavior (Baumeister and Vohs, 2016). Thus, they are more impulsive than under normal state. Ego depletion is detrimental because ego-depleted individuals are highly likely to demonstrate unethical behavior in the workplace (Trevino et al., 2014). Given that unethical behavior seriously impairs the benefits of organizations, numerous studies have explored the relationship between ego depletion and unethical behavior in the workplace (e.g., Rosen et al., 2016; Yam et al., 2016; Klotz et al., 2018).

However, previous ego-depletion studies have mainly focused on the intrapersonal influences of ego depletion of individuals on their subsequent behavior, neglecting the interpersonal influences of ego depletion on the behavior of other individuals. Previous studies have overlooked the possible crossover effect of ego depletion. When the ego depletion state is transmitted from one person to another, it is highly possible to witness such interpersonal influence in that ego depletion of one person manifests its detrimental effect of inducing unethical behavior of other person. Unethical behavior refers to actions that exhibit harmful effects on others and is “either illegal or morally unacceptable to the larger community” (Jones, 1991, p. 367). Studies about ego depletion in the workplace have mainly viewed ego depletion as a mediating mechanism through which personal and/or contextual factors result in unethical behavior of employees. For example, Christian and Ellis (2011) found that poor sleep quality among individuals depleted their self-control resources, thereby adding deviant behavior in the workplace. Yam et al. (2016) found that surface acting of supervisors depleted their self-control resources, which increased abusive supervision. This approach of exploring the intrapersonal relationship between ego depletion and unethical behavior in the workplace stemmed from the dual-task paradigm of ego depletion studies in the social psychology area, which contained two unrelated self-control tasks (Converse and DeShon, 2009). The first self-control task (independent variable) depletes self-control resources of participants; thus, they will lack sufficient self-control resources to perform well in the second self-control task (dependent variable). However, neglecting the possible interpersonal effects of ego depletion leads to a limitation of the dual-task paradigm. In particular, the underlining assumption of this paradigm is that as one’s own undesirable cognitive state, ego depletion merely influences outcomes of the depleted individual but not of others. Crossover literature has argued that several negative experiences, such as negative affect, depression, or emotion exhaustion, could be transmitted from leaders to their subordinates (Hoobler and Hu, 2013; Ten Brummelhuis et al., 2014; Li et al., 2016). This notion may imply that ego depletion, which is a type of negative experience, can also be transmitted from leaders to their subordinates, thereby inducing unethical behavior among its recipients. The present study employs the crossover perspective to explore possible interpersonal effects of ego depletion.

**FIGURE 1:**
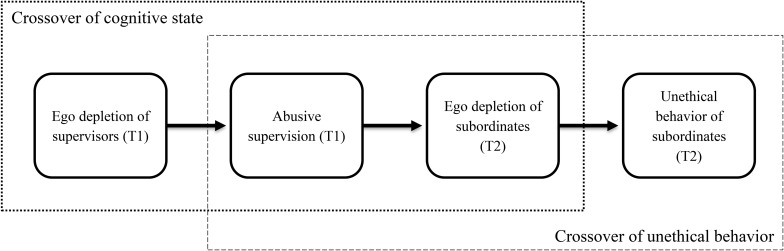
Theoretical model of this study.

In the organizational context, attitudes and behaviors of employees are more influenced by their supervisors than by their colleagues at the same level of hierarchy, because supervisors exhibit formal authority to make important decisions that are related to promotions, salary, or training opportunities of subordinates (Li et al., 2016). Thus, the crossover is more likely to occur from supervisors to subordinates (Ten Brummelhuis et al., 2014; Li et al., 2016). Relying on the crossover model (Westman, 2001; Bakker et al., 2009) and the conservation of resources (COR) theory (Hobfoll, 1989, 2001; Hobfoll et al., 2018), we propose that ego depletion could influence unethical behavior interpersonally. Crossover model posits an indirect crossover process in which the interpersonal influence plays an important role for transmitting negative experiences from supervisors to their subordinates (Westman, 2001). COR theory argues that individuals under a state of resource depletion will exert effort to protect their remaining resources against further loss (Hobfoll, 2001). Moreover, integrating these two theories, a serial crossover model is developed to explain how the crossover of ego depletion and the crossover of unethical behavior are entwined, thereby inducing the interpersonal influence of ego depletion. This newly developed serial crossover model (see Figure 1) demonstrates that depleted supervisors will exhibit abusive supervision (Courtright et al., 2016), thereby depleting self-control resources of subordinates (Thau and Mitchell, 2010); to protect their remaining resources, subordinates are reluctant to exercise self-regulation and are prone to impulsive behavior (Deng et al., 2018). As a result of this serial crossover, subordinates of those ego-depleted supervisors are prone to exhibit deviant behaviors.

The present study contributes to the COR and crossover literatures in several ways. First, by combining the crossover model and the COR theory, we propose a serial crossover model that helps understand why and how supervisors’ ego depletion leads to subordinates’ deviant behavior. Extant ego depletion studies following the dual-task paradigm mainly focus on the intrapersonal effects of ego depletion. This paradigm stresses that resource depletion can merely influence the behavior of depleted individuals. Our current study incorporates the crossover perspective and proposes that self-control resource depletion can be transmitted from one person to another, thereby inducing deviant behavior of another individual. This helps broaden our understanding of the interpersonal effect of self-control resource depletion. Second, our proposed serial crossover model sheds lights on the multiple crossover processes consisting of transmissions of both ego depletion and unethical behavior from supervisors to their subordinates. While the crossover literature has limited the focus on the crossover of either negative state or unethical behavior (e.g., Ten Brummelhuis et al., 2014; Li et al., 2016), our model highlights that the transmissions of ego depletion and unethical behavior are entwined rather than independent with each other. Thus, our current study extends the scope of crossover research to simultaneously examine transmissions of these two related negative states. Third, different from most existing crossover studies that limit their focuses on the transmission of affective states (e.g., burnout, emotional exhaustion, and anxiety), we propose that crossover can happen for other states, the cognitive state of ego depletion in the current study as an example. By doing so, our study enriches the content of the crossover model.

### Theories and Hypotheses

#### Crossover of Ego Depletion in the Workplace

Crossover refers to the process whereby job stressors or psychological strains are transmitted from one individual to other people (Westman, 2001; Bakker et al., 2009). The stressor/strain crossover process includes three mechanisms, namely, the direct empathetic crossover, the spurious effect from common stressors, and the indirect process (Westman, 2001). Direct process refers to the empathetic reaction of stress recipients to stress donors; thus, recipients will feel the stress of donors. The spurious effect from common stressors refers to certain life events that induce stress between both partners. For example, the unemployment experience of a husband or a wife will inevitably elicit the stress of his/her spouse. The indirect process posits that the crossover process has certain mediators such as social support, social undermining, and coping strategies. It needs to point out that previous studies have mainly focused on the direct empathetic crossover process of several negative affective states (Westman, 2001; Bakker et al., 2009; Ten Brummelhuis et al., 2014; Li et al., 2016). This is probably due to the fact that most researchers are interested in the transmission of stresses, which are negative and emotion-related by nature. Someone’s negative emotional experiences, such as negative affect, depression, or emotion exhaustion, can be felt directly by someone else because individuals are sensitive to negative emotions of others. To our knowledge, only a few studies have explored the indirect crossover process in transmitting negative affective states (e.g., Ten Brummelhuis et al., 2014; Li et al., 2016).

However, indirect crossover process may be more important in understanding the transmission of negative cognitive state from supervisors to their subordinates in the workplace. Unlike the negative affective state, ego depletion is a type of negative cognitive state that can hardly be directly observed or felt by others. The transmission of ego depletion from supervisors to their subordinates may further rely on explicit behaviors of the former. Extant research has indicated that the state of self-control failure (i.e., ego depletion) can lead to the self-control failure behavior (e.g., unethical behavior) (Gino et al., 2011). The unethical behavior of ego-depleted supervisor may manifest in the form of abusive supervision, which is a type of social undermining (Barnes et al., 2015; Lin et al., 2016). Abusive supervision refers to “subordinates’ perceptions of the extent to which supervisors engage in the sustained display of hostile verbal and non-verbal behaviors, excluding physical contact” (Tepper, 2000, p. 178). Supervisors face numerous temptations to exert abusive behaviors toward their subordinates in the workplace (Barnes et al., 2015). For example, when a subordinate commits a mistake, the supervisor will have the impulse to belittle this subordinate. Furthermore, engaging in abusive supervisory behavior can exhibit immediate benefits for supervisors. Such benefits include improved recovery level, which refers to “the extent to which the negative consequences of short-term strain reactions are reduced and individuals are brought back to their pre-stressor level of functioning” (Qin et al., 2018, p. 1952). Supervisors who face the stress of self-control resource depletion will have numerous opportunities to display impulsive abusive behaviors toward their subordinates given that they hold high positions. COR theory states that individuals will strive to obtain, retain, and protect resources (Hobfoll, 2001). Furthermore, resource loss is more salient than resource gain. Under the state of resource depletion, individuals will attempt to protect their resources from further loss. As previously mentioned, supervisors face many temptations to exert abusive supervision toward their subordinates. Under the state of ego depletion, they have little resources to inhibit themselves from doing the abusive behavior. Moreover, neither are they motivated to use their remaining self-control resources to control themselves to prevent the further loss of their limited resources. Empirical evidence also supports that supervisors’ ego depletion is positively related to abusive supervision (e.g., Barnes et al., 2015; Lin et al., 2016).

Abusive supervision involves ridiculing, undermining, and yelling to subordinates, which are salient workplace stressors that threaten the actual or potential loss of valued resources of the latter (Xu et al., 2015). Being abusively treated, subordinates will experience strong negative affect (Hoobler and Hu, 2013). Lian et al. (2014) cautioned that they would even retaliate their abusive supervisors. However, supervisors are normally more powerful than subordinates in an organization, and can yield substantial influences on the employment security and career opportunities of their subordinates. Therefore, even when abused by their supervisors, the rational reaction of subordinates is to suppress their negative emotion or vindictive intention toward their supervisors. Unfortunately, this volitional process will deplete self-control resources of subordinates (Baumeister and Vohs, 2016). Preliminary study has provided empirical evidence to demonstrate that abusive supervision depletes self-control resources of subordinates (e.g., Thau and Mitchell, 2010).

In summary, supervisors under the state of ego depletion are more likely to adopt abusive supervision toward their subordinates because they lack self-control resources to resist the temptation of doing so. Subsequently, subordinates who suffer from abusive supervision are likely to fall into the state of ego depletion. Therefore, we propose an indirect crossover process through which the negative cognitive state is transmitted from supervisors to subordinates via this social undermining mechanism.

*Hypothesis 1*: Abusive supervision mediates the relationship between supervisors’ ego depletion and subordinates’ ego depletion.

#### Crossover of Unethical Behavior in the Workplace

Drawing on the crossover model, we further propose that unethical behavior can be transmitted from supervisors to subordinates. Workplace unethical behavior (deviant behavior) is defined as “voluntary behavior that violates significant organizational norms, and in so doing, threatens the well-being of the organization and/or its members” (Robinson and Bennett, 1995, p. 556). Unethical behavior in the workplace involves two dimensions, namely, moral intensity and directivity (Robinson and Bennett, 1995). Moral intensity refers to the extent of issue-related moral imperative under a situation (Jones, 1991). In terms of unethical behavior in the workplace, moral intensity defines whether the unethical behavior is minor or serious. Directivity dimension indicates whether the victim of workplace unethical behavior is directing to the interpersonal or the organizational (Robinson and Bennett, 1995). Based on these two dimensions, workplace unethical behavior is categorized into four types, namely, production deviance, property deviance, political deviance, and personal aggression (Robinson and Bennett, 1995). Workplace unethical behavior can seriously harm organizations (Gino et al., 2011; Trevino et al., 2014). For example, workplace unethical behaviors among employees such as theft of office supplies, fraudulent expense reports or injury claims, and falsified overtime, are costing US companies an estimated 50 billion dollars annually (see Weber et al., 1999; Mishra and Prasad, 2006). What is more, workplace unethical behavior is usually transmitted from one person to another, particularly from supervisors to their subordinates (Zhang et al., 2019).

Abusive supervision is the personal aggression of supervisors toward their subordinates (Tepper, 2000). As previously mentioned, abusive supervision can deplete self-control resources of subordinates. Moreover, subordinates’ ego depletion may also lead to their own unethical behavior. When individuals are faced with the temptation to do workplace unethical behavior, two forces influence their behavior. On the one hand, unethical behavior may elicit short-term benefits such as exacting revenge to the abusive supervisor; on the other hand, unethical behavior may impair long-term benefits such as acquiring good career opportunities (Yam et al., 2014). Individuals often want to obtain short-term benefits through unethical behavior, but they also want to maintain their long-term moral image to gain recognition from their supervisors (Gino et al., 2011). A trade-off between the short-term and long-term benefits emerges when individuals face ethical decisions. In order to achieve long-term benefits, individuals rely on self-control resources to inhibit their desires to satisfy short-term benefits. COR theory states that individuals will exhibit impulsive behavior to protect their remaining resources when their valued resources are depleted, which leads to loss spirals (Hobfoll et al., 2018). Impulsive behaviors are characterized by the exchange of short-term benefits at the expense of long-term benefits, including deviant behavior (Yam et al., 2014). Empirical evidence has revealed that ego depletion can elicit unethical behavior in the workplace (e.g., Christian and Ellis, 2011; Deng et al., 2017; Klotz et al., 2018). Thus, we predict the following:

*Hypothesis 2*: Subordinates’ ego depletion mediates the relationship between abusive supervision and subordinates’ deviant behavior.

#### Serial Crossover of Ego Depletion and Unethical Behavior

Based on the fore-mentioned argument, we propose a serial crossover process that consists of transmissions of both negative cognitive state and workplace deviant behavior from supervisors to their subordinates. More specifically, this serial crossover model posits that while abusive supervision mediates the effect of supervisors’ ego depletion on subordinates’ ego depletion, the latter further conveys the effect of abusive supervision on subordinates’ unethical behavior.

Notably, such a serial crossover is the consequence of simultaneous self-control failures of supervisors and their subordinates. Supervisors face many temptations to exert abusive behaviors toward their subordinates (Barnes et al., 2015; Qin et al., 2018). COR theory (Hobfoll, 1989, 2001; Hobfoll et al., 2018) posits that when self-control resources of supervisors are depleted, they become impulsive to show abusive behavior to protect their limited resources. Recipients of abusive supervision, namely, the subordinates, will experience huge psychological stress, thereby depleting self-control resources of subordinates. This process depicts the crossover of negative cognitive state (i.e., ego depletion) from supervisors to their subordinates.

Moreover, subordinates rely on self-control resources to make ethical decisions (Gino et al., 2011). Similar to their supervisors, subordinates under the ego-depleted state become impulsive and are unable to inhibit themselves from performing deviant behavior. Thus, unethical behavior is transmitted from supervisors to their subordinates, and subordinates’ ego depletion plays the role of mediating mechanism for such transmission. This process depicts the crossover of unethical behavior from supervisors to their subordinates.

Taking together, this serial crossover model delineates self-control failures of both supervisors and their subordinates. It should be noted that supervisors’ self-control failure is the cause of that of their subordinates. By combining these two crossover processes, we propose our final hypothesis.

*Hypothesis 3*: The effect of supervisors’ ego depletion on subordinates’ deviant behavior is serially mediated by abusive supervision of the former and ego depletion of the latter.

## Materials and Methods

### Participants and Procedures

We obtained the survey data from supervisors and their subordinates in a big call center of a state-owned insurance company in China. The sampling process was supported by the human resource management department. We collected the data in two waves to minimize the common method variance. During the first wave, we collected data pertaining to supervisors’ ego depletion, subordinates’ perception of abusive supervision, and demographic variables of both supervisors and subordinates. Six weeks after the first wave, we conducted a second survey to obtain measures of subordinates’ ego depletion and subordinates’ deviant behavior. Ten Brummelhuis et al. (2014) cautioned that in deciding the interval length for a multi-wave survey study, researchers should take into account the potential effect of sudden or unexpected life events, which might happen during two waves, on variables measured in the later wave. Hence, they suggested that a relatively short time interval was appropriate to avoid potential bias of unexpected life events. After reviewing the common strategy adopted in relevant extant studies and discussing the logistic issue with the manager of the company where our survey was conducted, we found it was feasible to conduct two surveys with an interval of 6 weeks. We intended to investigate 50 supervisors and their 500 subordinates. A total of 45 supervisors (90%) and 408 subordinates (81.6%) completed the first survey questionnaires. During the second wave, 192 subordinates completed their questionnaires (47.06%). The matching results of the two-wave survey questionnaires indicated that a total of 192 subordinates who participated in both surveys were under 24 supervisors. On average, each supervisor had 8 subordinates.

Among the 24 supervisors, 20 were male, and 4 were female; 15 supervisors held an associate degree, and 9 others with a bachelor degree. Among the 192 subordinates, 107 were male (55.7%); the mean age was 25.89 (*SD* = 3.95) (five subordinates failed to report their age information); 147 subordinates acquired an associate degree or above (76.6%), 44 subordinates had a high school degree or below (22.9%), and 1 subordinate failed to report his/her education information (0.5%). On average, these subordinates had been in this company for 3.72 years (*SD* = 3.03).

To verify whether there was any systematic bias due to the drop-out of subordinates in the second wave, we tested the differences of the demographic information of the remaining subordinates and those absent. Results revealed no differences on the sex (χ^2^_(__1__)_ = 3.51, ns), age (*t* = 1.38, ns), tenure (*t* = 0.94, ns), and education (χ^2^_ (__1__)_ = 0.79, ns) of these two categories of subordinates. These results indicated that no systematic bias was found between these two samples.

### Measures

#### Ego Depletion

We used the 25-item scale from Twenge et al. (2004) to measure ego depletion of supervisors and subordinates. This scale was used in previous studies and showed good reliability and validity (e.g., Deng et al., 2017). Sample items were “I want to give up,” and “I feel like my willpower is gone.” Supervisors and subordinates were asked to rate their feelings during the past week on a scale ranging from 1 (not true) to 7 (very true). The Cronbach’s α of this scale for the supervisor sample was 0.92, whereas the Cronbach’s α for the subordinate sample was 0.89.

#### Abusive Supervision

We used the 10-item abusive supervision scale from Aryee et al. (2008) to measure the subordinates’ perception of abusive supervision. This scale was adapted from the scale of Tepper (2000) and revealed good reliability and validity in the Chinese context. Sample items were “My supervisor gives me the silent treatment,” “My supervisor reminds me of my past mistakes and failures,” and “My supervisor makes negative comments about me to others.” Response options ranged from 1 (never) to 5 (always). The Cronbach’s α of this scale in our study was 0.83.

#### Deviant Behavior

We used the 17-item workplace deviant behavior scale from Newstrom and Ruch (1975) to measure subordinates’ deviant behavior. This scale had been used in previous studies and showed good reliability and validity (e.g., Zheng et al., 2019). We found that three items of the scale failed to fit our sample in the early interview with employees in the call center^1^ (i.e., “Authorizing a subordinate to violate company rules,” “Padding an expense account up to 10%,” and “Padding an expense account more than 10%”). Thus, we dropped these three items and only used the remaining 14 items to measure subordinates’ deviant behavior. As admitting doing deviant behavior posted a threaten to the self-image of subordinates, the measuring of deviant behavior was found to be sensitive to the social desirability response bias (Yang et al., 2017). Moreover, we collected data in a call center where employees were working in the same small space with their supervisor and colleagues, and employees were also closely monitored by their supervisors. Under this circumstance, it was unlikely for them to consciously report any deviant behavior if asked directly. Therefore, we believed it was necessary to take precautionary measures of mitigating the social desirability tendency of respondents. Accumulative evidence in behavioral ethics literatures had indicated that the indirect questioning method could help reduce the social desirability response bias in measuring construct prone to social influences (Fisher, 1993), the measurement of deviant behavior as an example (Yang et al., 2017). We employed this technique in the current study to reduce the social desirability bias in measuring subordinates’ deviant behavior. Specifically, participants were asked to report “How often have you observed the following types of behavior in your organization?” This method had been widely used in extant studies to reduce the social desirability response bias in measuring unethical behavior (e.g., Treviño and Weaver, 2001; Beekun et al., 2010; Bossuyt and Van Kenhove, 2018). Sample items were “Use company service for personal use” and “Claim credit for someone else’s work.” Response options ranged from 1 (never) to 5 (very frequently). The Cronbach’s α of this scale in our study was 0.98.

#### Control Variables

We controlled the sex, age, tenure, and education of subordinates for the possible influences of these demographic variables in the crossover process. For example, stress recipients’ sex was perceived as an important factor for influencing the crossover process (Westman, 2001).

## Results

### Preliminary Analyses

Prior to testing our hypotheses, we conducted a confirmatory factor analysis (CFA) to assess the discriminate validity of variables from subordinates. Following the recommendation of Hall et al. (1999), we parceled the items within each scale to serve as indicators of the latent variable when the number of items for the variable exceeded three. We used the item-to-construct balance method to parcel the items (Little et al., 2002). Prior studies have recommended creating three parcels for latent variables with few items and more than three parcels for latent variables with copious items (cf., Carlson et al., 2012). Thus, we created three parcels for the 10-item measure of abusive supervision, five parcels for the 25-item scale of subordinates’ ego depletion, and three parcels for the 14-item measure of subordinates’ deviant behavior. Results demonstrated good model fit for the three-factor model on the data of subordinates (χ*^2^/df* = 2.27, NFI = 0.96, CFI = 0.98, GFI = 0.92, RMSEA = 0.082, SRMR = 0.043), which was better than other competitive models (see Table 1).

**TABLE 1:** Confirmatory factor analyses of the data from subordinates.

Models	χ^2^	df	Δ (χ 2(Δ df))	CFI	TLI	RMSEA	SRMR
Model 1 (three-factor model): abusive supervision, subordinates’ ego depletion, deviant behavior subordinates	93.13***	41	/	0.98	0.97	0.082	0.043
Model 2a (two-factors model): abusive supervision and subordinates’ ego depletion combined	363.02***	43	269.89 (2)***	0.81	0.76	0.197	0.139
Model 2b (two-factor model): subordinates’ ego depletion and subordinates’ deviant behavior combined	882.13***	43	789 (2)***	0.51	0.37	0.320	0.173
Model 2c (two-factor model): abusive supervision and subordinates’ deviant behavior combined	385.78***	43	292.65 (2)***	0.84	0.79	0.204	0.163
Model 3 (single factor model)	1260.92***	44	1167.79 (3)***	0.42	0.28	0.381	0.221

***p < 0.001 (two-tailed tests).

### Descriptive Statistics

Table 2 indicates the means and standard deviations of and correlations among variables in this study. Internal consistency reliabilities, when available, were reported along the diagonal. Table 2 reveals that supervisors’ ego depletion is positively related to abusive supervision (*r* = 0.24, *p* < 0.001); abusive supervision is positively related to subordinates’ ego depletion (*r* = 0.22, *p* < 0.01); and subordinates’ ego depletion is positively related to subordinates’ deviant behavior (*r* = 0.30, *p* < 0.001). The pattern of these correlations provides initial support for our serial mediation hypothesis.

**TABLE 2:** Means, standard deviations, and correlations among study variables.

	*M*	*SD*	1	2	3	4	5	6	7	8
**Level 1 variables**										
(1) Sex	0.56	0.50	/							
(2) Age	25.89	3.95	–0.13	/						
(3) Education	2.88	0.59	0.02	0.14*	/					
(4) Tenure	3.72	3.03	−0.18*	0.52***	−0.05	/				
(5) Abusive supervision	1.51	0.52	0.11	−0.09	−0.05	−0.09	(0.83)			
(6) Subordinates’ ego depletion	3.14	0.91	–0.10	−0.12	0.00	−0.19**	0.22**	(0.89)		
(7) Subordinates’ deviant behavior	1.26	0.63	–0.01	−0.03	−0.14	−0.07	0.15*	0.30***	(0.98)	
**Level 2 variables**										
(8) Supervisors’ ego depletion	5.36	0.60	0.12	−0.28***	−0.08	−0.21**	0.24***	0.04	0.14	(0.89)

N = 192 at the subordinate level, and N = 24 at the supervisor level. Data for Variables 1–7 were reported by subordinates; Variable 8 was reported by supervisors. For gender, 0 = female, 1 = male. For education, 1 = junior middle school or lower, 2 = high school, senior high school, or technical school, 3 = associate degree, 4 = bachelor degree, 5 = master’s degree, 6 = doctoral degree. Scores of variable 8 is disaggregated to the subordinates’ level to calculate correlations. Reliability estimates are reported in parentheses along the main diagonal. *p < 0.05; **p < 0.01; ***p < 0.001 (two-tailed tests).

### Tests of Hypotheses

Our data contain a hierarchical structure in which measures of individual-level variables are nested among supervisors. In addition, two out of three hypotheses are multilevel in nature and involve the testing effects of supervisor-level variables (Level 2) on individual-level variables (Level 1). To appropriately demonstrate this effect, we used multilevel path analysis to simultaneously estimate the hypothesized multilevel relationships using Mplus 7.0 (Muthén and Muthén, 1998–2013). We followed the recommendations of Preacher et al. (2010) to test our multilevel serial mediation model. Prior to testing the hypotheses, we examined whether significant between-person variances existed for Level 1 variables. The null model specified abusive supervision, subordinates’ ego depletion, and subordinates’ deviant behavior as outcome variables and included no predictors at either Level 1 or Level 2 to examine the between-person variances. Results showed that the intraclass correlation (ICC1) values for these three measures are 0.14, 0.11, and 0.10, respectively (*F* = 11.29–27.48, all *p* < 0.01). As these values suggest that there are substantial variances at Level-2 for all three individual-level variables, multilevel analysis is needed (LeBreton and Senter, 2008).

Table 3 presents the multilevel modeling results. After demographic variables are controlled, results reveal that supervisors’ ego depletion is positively related to abusive supervision (γ = 0.24, *t* = 2.01, *p* < 0.05). After demographic variables and supervisors’ ego depletion are controlled, abusive supervision is positively related to subordinates’ ego depletion (γ = 0.35, *t* = 2.50, *p* < 0.05). After controlling demographic variables, supervisors’ ego depletion, and abusive supervision; subordinates’ ego depletion is positively related to subordinates’ deviant behavior (γ = 0.19, *t* = 2.37, *p* < 0.05).

**TABLE 3:** Multilevel results of the relationship between supervisors’ ego depletion and subordinates’ deviant behavior through abusive supervision and subordinates’ ego depletion.

Variables	Abusive supervision		Subordinates’ ego depletion		Subordinates’ deviant behavior	
	*Est.*	*SE*	*Est.*	*SE*	*Est.*	*SE*
**Level 1**						
Sex	0.36	1.17	–1.19	3.28	–2.92	4.12
Age	0.15	0.08	0.19	0.69	0.02	0.12
Education	–0.32	0.71	0.13	3.76	2.49	3.51
Tenure	–0.18	0.17	–0.66	1.36	–0.27	0.24
Abusive supervision			0.35*	0.14	0.07	0.13
Subordinates’ ego depletion					0.19*	0.08
**Level 2**						
Intercepts	–2.18	1.93	1.06	5.80	–5.31	5.92
Supervisors’ ego depletion	0.24*	0.12	–0.14	0.48	0.14	0.11

*p < 0.05 (two-tailed tests).

Table 4 presents the results of multilevel serial mediation analyses. Results reveal that abusive supervision mediates the relationship between supervisors’ ego depletion and subordinates’ ego depletion (a1 × d1 = 0.083, *SE* = 0.028, *p* < 0.01). The 95% confidence interval is [0.028, 0.138], which excludes zero, thereby supporting hypothesis 1. In addition, subordinates’ ego depletion mediates the relationship between abusive supervision and subordinates’ deviant behavior (d1 × b2 = 0.065, *SE* = 0.028, *p* < 0.05). The 95% confidence interval is [0.010, 0.121], which excludes zero, thereby supporting hypothesis 2. Hypothesis 3 indicates that abusive supervision and subordinates’ ego depletion serially mediate the relationship between supervisors’ ego depletion and subordinates’ deviant behavior. A formal test of the serial indirect effect reveals a statistically significant serial indirect effect of supervisors’ ego depletion on subordinates’ deviant behavior via abusive supervision and ego depletion of the latter (a1 × d1 × b2 = 0.016, *SE* = 0.007, *p* < 0.05). The 95% confidence interval is [0.001, 0.030], which excludes zero. Therefore, the serial indirect effect (Hypothesis 3) is supported.

**TABLE 4:** The results of mediation analyses.

Indirect effects	*Est.*	*SE*	95% CI
Hypotheses testing			
Supervisors’ ego depletion → Abusive supervision → Subordinates’ ego depletion (Hypothesis 1)	0.083	0.028	[0.028, 0.138]
Abusive supervision → Subordinates’ ego depletion → Subordinates’ deviant behavior (Hypothesis 2)	0.065	0.028	[0.010, 0.121]
Supervisors’ ego depletion → Abusive supervision → Subordinates’ ego depletion → Subordinates’ deviant behavior (Hypothesis 3)	0.016	0.007	[0.001, 0.030]
Supplemental analyses			
Supervisors’ ego depletion → Abusive supervision → Subordinates’ deviant behavior	0.016	0.028	[−0.039, 0.071]
Supervisors’ ego depletion → Subordinates’ ego depletion → Subordinates’ deviant behavior	−0.025	0.092	[−0.206, 0.155]

### Supplemental Analyses

We performed supplemental analyses to examine whether the hypothesized serial mediation was indispensable. Specifically, we calculated and tested two indirect effects from supervisors’ ego depletion to subordinates’ deviant behavior that only involved one-stage mediation. Table 4 reports the results of the supplemental analyses. For the first one, subordinates’ ego depletion fails to mediate the effect of supervisors’ ego depletion on subordinates’ deviant behavior (a2 × b2 = −0.025, *SE* = 0.092, *p* > 0.05). The 95% confidence interval is [−0.206, 0.155], which includes zero. For the second one, abusive supervision also fails to mediate such effect (a1 × b1 = 0.016, *SE* = 0.028, *p* > 0.05). The 95% confidence interval is [−0.039, 0.071], which includes zero.

Given that the serial mediation is supported but not the two one-stage mediations, it can be inferred that each form of crossover (i.e., the transmission of ego depletion or the transmission of unethical behavior from supervisors to their subordinates) is necessary in establishing the link between supervisors’ ego depletion and subordinates’ deviant behavior. In the other words, such effect would not be revealed without considering each form of crossover. In this sense, supplemental analyses provide additional evidence for our serial crossover model.

## Discussion

Integrating the crossover model and COR theory, we developed a serial crossover model to explain why and how supervisors’ ego depletion induced subordinates’ deviant behavior. The essential in this model is that it consists of both the cognitive state crossover and the unethical behavior crossover from supervisors to their subordinates. Our serial crossover model is characterized of three features. First, it captures the isomorphic self-regulation failure of supervisors and their subordinates. They are regarded as isomorphic because self-regulation failures manifest in the same form for supervisors and their subordinates. More specifically, unethical behaviors of the both sides are the consequences of the shortage of self-regulating resources. Second, our serial crossover model indicates that these two processes of self-regulation failures are not independent but entwined with each other. The self-regulation failure of subordinates is the result of that of their supervisors. Finally, this newly-constructed model demonstrates that supervisors’ personal state can yield substantial influences on subordinates’ explicit behavior through, and only through, the serial crossover processes.

### Theoretical Contributions

Our study provides several important theoretical contributions to the COR and crossover literatures. First, our study contributes to the COR theory through introducing the interpersonal perspective for explaining why and how the crossover of self-control resource depletion can occur among close working partners. Previous studies on the COR theory have mainly focused on the intrapersonal consequences of resource depletion (e.g., resource loss spiral for the focal individual) (Hobfoll et al., 2018), neglecting the fact that resource depletion can also be transmitted from one person to another. For example, Deng et al. (2018) found that resource depleted employees would engage in more harmful behaviors toward others than those who do not experience resource depletion. However, whether an individual under the resource depletion state can influence the state/behavior of another should be tackled. By combining the crossover model and COR theory, we found that ego depletion could be transmitted from supervisors to their subordinates through abusive supervision, thereby inducing subordinates’ unethical behavior. Following the tradition of dual-task paradigm of ego depletion studies, previous studies have merely explored the intrapersonal relationship between ego depletion and unethical behavior. Previous studies have neglected the interpersonal relationship between ego depletion and unethical behavior. By combining the crossover model and COR theory, we assumed and found the serial crossover of supervisors’ ego depletion on subordinates’ unethical behavior. This finding extends the scope of COR theory from intrapersonal to interpersonal. In addition, the current study offers an interpersonal perspective in exploring the consequences of resource depletion. For example, previous studies have found a positive intrapersonal relationship between ego depletion and negative affect (Furley et al., 2019). Given that negative affect is usually transmitted from one person to another through the empathetic process, future study can explore whether ego depletion of an individual can elicit negative affect of others.

Second, our study enriches the content of crossover model through revealing the crossover process of ego depletion, a negative cognitive state that is largely neglected in the literature. The crossover model was developed to describe the crossover of stress from one person to another (Westman, 2001). However, most studies adopting this model have mainly focused on the crossover of affective states, such as depression, anxiety, emotion exhaustion, and distress (Bakker et al., 2009). To the best of our knowledge, the current study is the first to investigate whether the cognitive state can be transmitted from one person to another. By combining the crossover model and COR theory, we found that self-control resource depletion could be transmitted from supervisors to their subordinates. When self-control resources of supervisors were depleted, they could not prevent themselves from engaging in the social undermining behavior. Furthermore, our study contributes to the crossover model by revealing the mechanism of the crossover of cognitive state. Specifically, it is the indirect social undermining process, but not the direct empathetic process, that transmitting ego depletion from supervisors to subordinates (Westman, 2001).

Third, the serial crossover model proposes a novel perspective to describe how team-level self-control failure, including team ego depletion and team unethical climate, can be developed through interpersonal processes. In this study, we found the crossover phenomenon of the isomorphic self-control failures from supervisors to their subordinates for the first time. The consequence of self-control failure was the same for supervisor and their subordinates in that both of them committed unethical behavior when under the ego-depleted state. As a result, negative cognitive state and unethical behavior will permeate to the whole team, giving rise to team ego depletion and team unethical climate. With regard to team ego depletion, few studies have studied the development processes of self-control failure state of a team. By combining the crossover model and COR theory, we found that ego depletion could be transmitted from supervisors to their subordinates. Therefore, team ego depletion may be developed from ego depletion of supervisors through the crossover processes. With regard to unethical climate of a team, previous studies have mainly focused on the influences of the cognitive moral development level among leaders in the formation of team (un)ethical climate (Trevino et al., 2014). Moreover, previous studies have mainly focused on the social learning process or social exchange process for leaders to influence ethical behavior of their subordinates (Brown and Treviño, 2006; Den Hartog, 2015). Our serial crossover model proposes that the crossover process acts as a new mechanism in forging team unethical climate. Specifically, our study indicates that unethical behavior is transmitted from supervisors to their subordinates, causing a similar behavioral pattern within the whole team. In this sense, unethical climate of a team may also be developed from unethical behavior of supervisors through the crossover process.

### Practical Implications

Our serial crossover model offers important implications on how to mitigate the vicious spiral of ego depletion/unethical behavior in the workplace. First, our serial mediation model indicates that all the negative consequences start with the ego depletion state. Supervisors under the ego-depleted state tend to behave unethically toward their subordinates in the first place. Moreover, this negative cognitive state can be transmitted from supervisor to their subordinates, thereby inducing unethical behaviors among the latter. Thus, organizations should develop a reasonable system to prevent ego depletion. For example, several studies have found that the mindfulness training could enhance self-control capacity of individuals (Creswell, 2017). Introducing mindfulness training programs for employees in organizations would be feasible to prevent ego depletion. Furthermore, the rest time of employees should be ensured (particularly for managers); previous studies have found that self-control resources could be restored after a period of rest (Baumeister and Vohs, 2016).

Second, our model implies that the crossover linkage of ego depletion should be disconnected from supervisors to their subordinates. We found that abusive supervision was the indirect process for transmitting ego depletion from supervisors to subordinates; thus, pertinent tactics should be developed to mitigate the relationship between supervisors’ ego depletion and abusive supervision or to mitigate the relationship between abusive supervision and subordinates’ ego depletion. Previous studies have found that moral identity could moderate the relationship between ego depletion and unethical behavior (Gino et al., 2011). That is, supervisors with high moral identity trait should be selected because they can reduce abusive behaviors toward their subordinates when they are depleted. We can also break the relationship between abusive supervision and subordinates’ ego depletion. For example, subordinates with strong self-control trait can mitigate the influences of abusive supervision (Yuan et al., 2020). Thus, organizations should hire employees with strong self-control trait.

### Limitations and Future Directions

This study exhibits several limitations. First, this study mainly focused on the crossover phenomenon of ego depletion and unethical behavior, but failed to pay attention to the possible boundary conditions under which these relationships could improve or reduce strength. For example, this study found that supervisors’ ego depletion was positively related to abusive supervision. Evidently, not all subordinates perceived their depleted supervisors as abusive supervisors. Therefore, several moderators may influence the relationship between supervisors’ ego depletion and abusive supervision. Future studies can explore boundary conditions when the crossover phenomenon in this study is stronger or weaker.

Second, similar to numerous previous crossover studies, this study explored negative experience crossover. We examined one type of non-emotion-related stress, namely, ego depletion, which was different from previous studies, but remained a negative experience. Bakker et al. (2009) argued that several positive experiences could also be transmitted from one person to another. Future studies can explore the crossover phenomenon of positive experiences, including work engagement. Their crossover mechanism can be different from negative experiences.

Finally, although we collected data during two waves and from two sources that helped reduce the common methods variance in survey research (Podsakoff et al., 2003), the relationships examined in the current study were still based on correlations. That is, this study cannot provide causal evidence for relationships among the variables in our serial crossover model. Several related studies may have demonstrated the causal relationship between ego depletion and unethical behavior (e.g., Gino et al., 2011); however, supervisors can be more likely to abuse their subordinates who have shown unethical behavior. Thus, future studies should employ a more rigorous research design (such as experimental or longitudinal design) than the one used in this study to test the causal relationships. What is more, ego depletion can be viewed as a relatively stable state or a momentary state (Baumeister and Vohs, 2016). By using the two-wave survey design, we might have caught the accumulative effects of supervisor’s behavior (i.e., abusive supervision) on subordinates’ state (i.e., subordinates’ ego depletion). However, this design is insufficiently in exploring the momentary effects of abusive supervision on subordinates’ ego depletion. We encourage researchers to use more dynamic approach, for example the daily-diary design, to explore the crossover effects of momentary state of ego depletion.

## Conclusion

In the workplace, it is common to see the crossover phenomenon that some negative experiences be transmitted from supervisors to their subordinates. By integrating the crossover model and COR theory, our current study indicates that both ego depletion and unethical behavior can be transmitted from supervisor to subordinates in the workplace. Furthermore, this study develops a serial crossover model to depict how the crossover of ego depletion and that of unethical behaviors are intertwined. By doing so, our study sheds new lights on the underlining mechanism of why supervisors’ ego depletion can lead to subordinates’ deviant behavior.

## Data Availability Statement

The datasets generated for this study are available on request to the corresponding author.

## Ethics Statement

The studies involving human participants were reviewed and approved by Institutional Review Board of the Institute of Psychology, Chinese Academy of Sciences. Written informed consent for participation was not required for this study in accordance with the national legislation and the institutional requirements (Protocol Number: H17002).

## Author Contributions

XM, XB, and LL designed this study. XM collected and analyzed the data. XM and XB wrote the manuscript.

## Conflict of Interest

The authors declare that the research was conducted in the absence of any commercial or financial relationships that could be construed as a potential conflict of interest.

## Footnotes

1 We deleted these items because the subordinates in this call center (employees at the bottom) lack the authority to pad their expenses and the authority to permit others to violate company rules.
